# Combination Treatment of IL‐17 Inhibitor and JAK Inhibitor in Psoriatic Arthritis: A Case Report and Scoping Review

**DOI:** 10.1002/iid3.70515

**Published:** 2026-09-11

**Authors:** Mengduan Pang, Ling Li, Qi Zhang, Min Zhao

**Affiliations:** ^1^ Department of Rheumatology and Immunology First Affiliated Hospital of Dalian Medical University Dalian China

**Keywords:** case report, IL‐17 inhibitor, JAK inhibitor, psoriatic arthritis, scoping review

## Abstract

**Background:**

Psoriatic arthritis (PsA) is a chronic inflammatory disease with complex pathophysiology. Despite advancements in biologic and targeted synthetic disease‐modifying antirheumatic drugs (b/tsDMARDs), treatment responses remain suboptimal for many patients.

**Methods:**

We present a case of a 35‐year‐old male with refractory PsA who achieved significant clinical improvement through combination therapy with ixekizumab (an IL‐17 inhibitor) and upadacitinib (a JAK inhibitor), following inadequate responses to multiple monotherapies. A scoping review of the literature was conducted, analyzing studies on dual b/tsDMARD therapy in PsA.

**Results:**

The patient achieved rapid clinical improvement with complete resolution of articular symptoms, normalization of inflammatory markers, and significant metabolic improvements. The scoping review identified 16 studies, highlighting the potential of multi‐target strategies while emphasizing safety concerns such as increased infection risks.

**Conclusion:**

This case and review underscore the promise of combination therapy in PsA while emphasizing the need for further research to optimize its use.

## 1 Introduction

Psoriatic arthritis (PsA) is a chronic inflammatory arthritis associated with psoriasis, characterized by heterogeneous clinical manifestations involving peripheral and axial joints, nails, skin, and entheses. This clinical variability frequently results in delayed diagnosis and treatment [1]. The pathogenesis of PsA is driven by a network of interconnected inflammatory cascades: activated dendritic cells and macrophages produce interleukin (IL)−23, which promotes T‐helper 17 (Th17) cell differentiation and sustains the IL‐17/IL‐22 axis [2]; concurrently, Janus kinase (JAK)‐signal transducer and activator of transcription (STAT) signaling serves as the critical intracellular conduit for multiple pro‐inflammatory cytokines, including type I/II interferons, IL‐6, and IL‐12/23 [3]. The convergence of these pathways on synovial fibroblasts, osteoclasts, and entheseal resident cells creates a self‐perpetuating inflammatory microenvironment that underlies joint damage, new bone formation, and associated metabolic comorbidities [4].

The advent of biologic and targeted synthetic disease‐modifying antirheumatic drugs (b/tsDMARDs) has significantly improved the management of PsA [5, 6]. However, despite an expanding therapeutic arsenal targeting diverse immunological pathways, no treatment trials have consistently achieved an ACR20 response rate exceeding 60% [5]. From an immunological perspective, this therapeutic ceiling reflects the redundancy and plasticity of inflammatory signaling: blockade of a single cytokine can be circumvented by compensatory upregulation of alternative pathways, sustained JAK‐STAT activation in tissue‐resident cells, and pathogenic contributions from innate immune cells independent of the targeted axis.

Recent studies suggest that dual cytokine inhibition ‐ achieved through either combining two biologic DMARDs (bDMARDs) or pairing a bDMARD with a targeted synthetic DMARD (tsDMARD)—may represent a promising therapeutic strategy for PsA [7]. By simultaneously targeting multiple inflammatory pathways, this approach addresses the disease's complex pathophysiology and enables broader suppression of inflammatory cascades [8]. Although limited clinical trials have explored dual biologic therapy in PsA, this strategy has yet to be adopted in clinical practice.

In this article, we report a clinical case of PsA successfully managed with combination therapy using ixekizumab (an IL‐17 inhibitor) and upadacitinib (a JAK inhibitor). Furthermore, we provide a comprehensive review of the scientific rationale and clinical evidence supporting the use of combined b/tsDMARDs in PsA treatment.

## 2 Methods

We conducted this scoping review in accordance with the methodological framework described by Arksey and O'Malley and the PRISMA‐ScR (Preferred Reporting Items for Systematic Reviews and Meta‐Analyzes extension for Scoping Reviews) guidelines [9]. The objective was to map the existing literature on combination b/tsDMARD therapy in PsA, identify the range of therapeutic combinations reported, and summarize clinical outcomes and safety data.

We systematically searched PubMed for English‐language studies published between January 1, 2000 and September 1, 2025 using the following MeSH terms: (1) “psoriatic arthritis” AND (“IL‐17 inhibitors” OR secukinumab OR ixekizumab OR brodalumab); (2) “psoriatic arthritis” AND (“TNF‐α inhibitors” OR infliximab OR adalimumab OR etanercept OR golimumab OR certolizumab pegol); (3) “psoriatic arthritis” AND (“JAK inhibitor” OR upadacitinib OR tofacitinib OR baricitinib); (4) “psoriatic arthritis” AND (“IL‐23 inhibitors” OR “IL‐12/23 inhibitors” OR guselkumab OR risankizumab OR ustekinumab OR tildrakizumab). The search strategy was developed by two authors (M.D.P. and M.Z.). No additional registers were searched.

**Figure 1:**
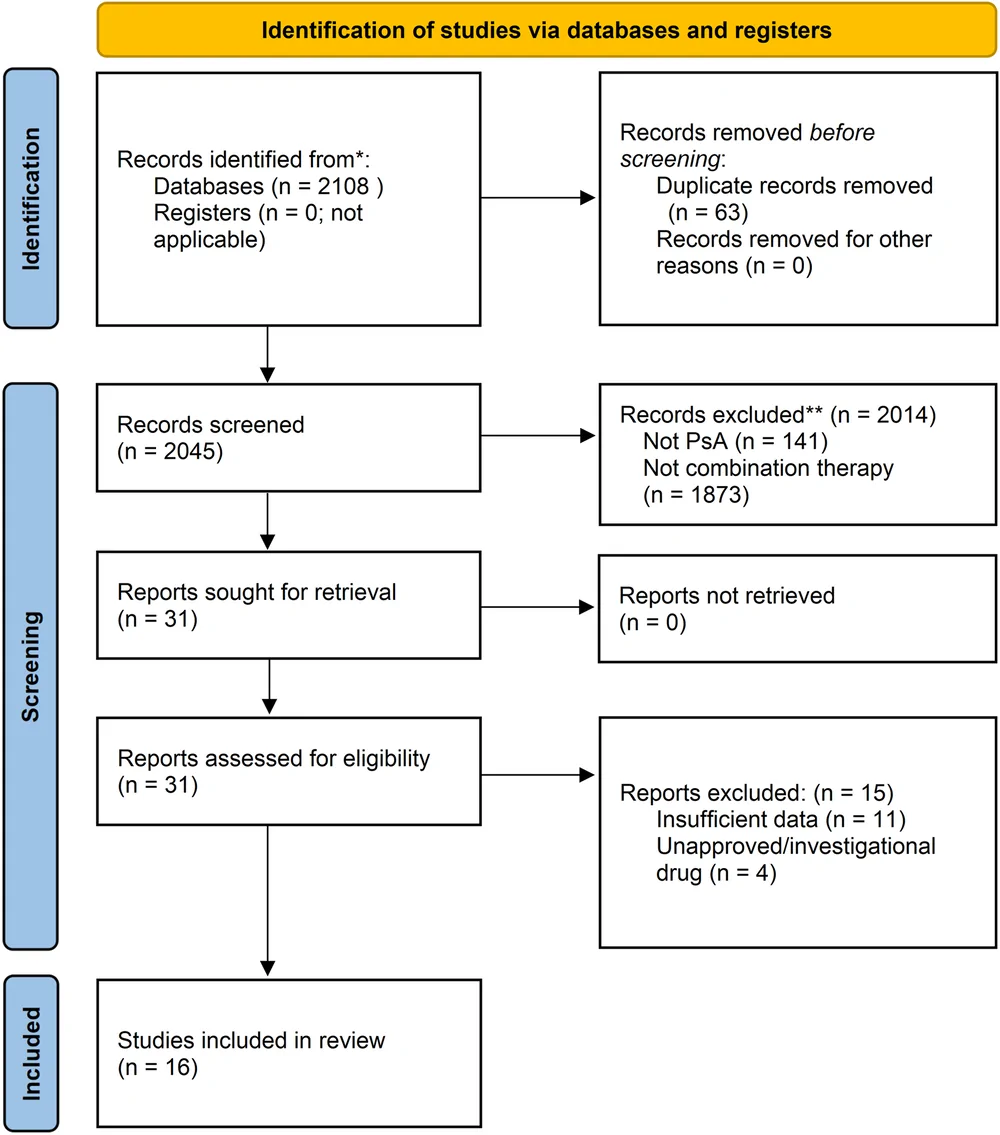
PRISMA‐ScR flow diagram. This scoping review adhered to the Preferred Reporting Items for Systematic Reviews and Meta‐Analyzes Extension for Scoping Reviews (PRISMA‐ScR) guidelines, and this flow diagram was created from the PRISMA 2020 flow diagram template. All screening and duplicate removal were performed manually. A total of 2108 records were identified from PubMed, and 2045 were screened after removing 63 duplicates. Sixteen studies were included that met our inclusion criteria. *PubMed was searched using MeSH terms for psoriatic arthritis combined with IL‐17 inhibitors, TNF‐α inhibitors, JAK inhibitors, and IL‐23/IL‐12/23 inhibitors. No additional registers were searched. **Records were excluded if they did not focus on psoriatic arthritis or did not report combination therapy with biologic or targeted synthetic DMARDs.

Two independent reviewers (M.D.P. and M.Z.) performed all screening and duplicate removal manually (Figure 1). Data were charted independently by two reviewers (M.D.P. and M.Z.) using a predefined extraction form, recording first author, year of publication, age/sex, treatment duration, joint involvement, combination regimen, clinical response, and adverse events. Given the predominance of case reports and small case series in the identified literature, and the heterogeneity of study designs and outcome measures, formal risk of bias assessment and meta‐analysis were not performed. This approach is consistent with the scoping review methodology, which prioritizes breadth of evidence mapping over critical appraisal and statistical synthesis. No review protocol was registered for this scoping review.

## 3 Case Presentation

Written informed consent was obtained from the patient for publication of this case report and any accompanying images. In April 2023, a 35‐year‐old male presented with progressive polyarthralgia involving the small joints of the hands, wrists, and bilateral hips, accompanied by chronic low back pain and morning stiffness lasting approximately 30 min. Previous treatment with sulfasalazine and diclofenac sodium had proven ineffective. The patient's body mass index (BMI) exceeded 30 kg/m^2^. His medical history included hypertension, hyperuricemia, hyperlipidemia, diabetes mellitus, and non‐alcoholic fatty liver disease.

The patient's erythrocyte sedimentation rate (ESR) was elevated at 49 mm/h (normal < 15 mm/h), and C‐reactive protein (CRP) levels were 29.4 mg/L (normal < 8 mg/L). Serological testing revealed negative results for rheumatoid factor, anti‐CCP antibodies, and HLA‐B27. Joint ultrasonography demonstrated effusion and synovial hyperplasia in the small joints of the hands and wrists.

A diagnosis of seronegative rheumatoid arthritis (RA) was established. Initial therapy included oral methotrexate combined with etanercept (25 mg subcutaneously every 2 weeks), which led to partial relief of joint pain. However, elevated alanine aminotransferase (ALT) levels prompted discontinuation of methotrexate due to suspected drug‐induced hepatotoxicity. Subsequently, etanercept monotherapy proved ineffective, and treatment was switched to adalimumab (40 mg subcutaneously every 2 weeks). Despite this adjustment, therapeutic response remained suboptimal.

**Figure 2:**
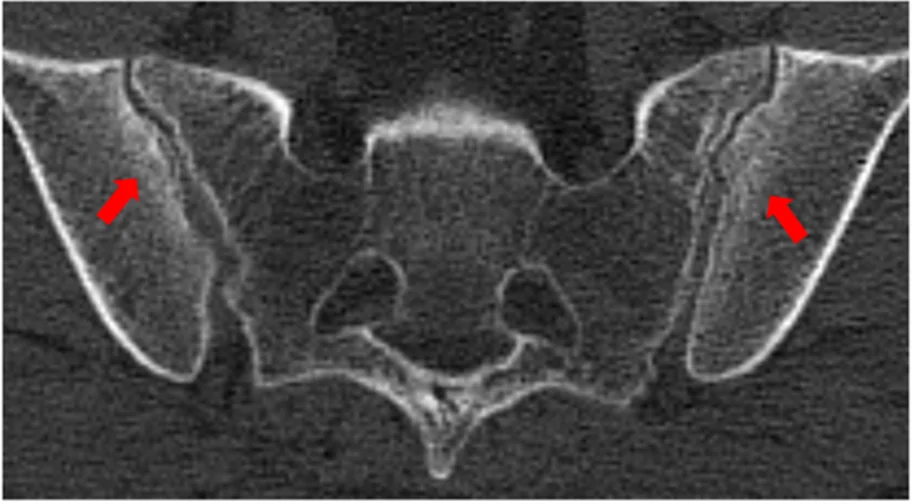
CT imaging demonstrated bilateral sacroiliitis. (arrows).

**Figure 3:**
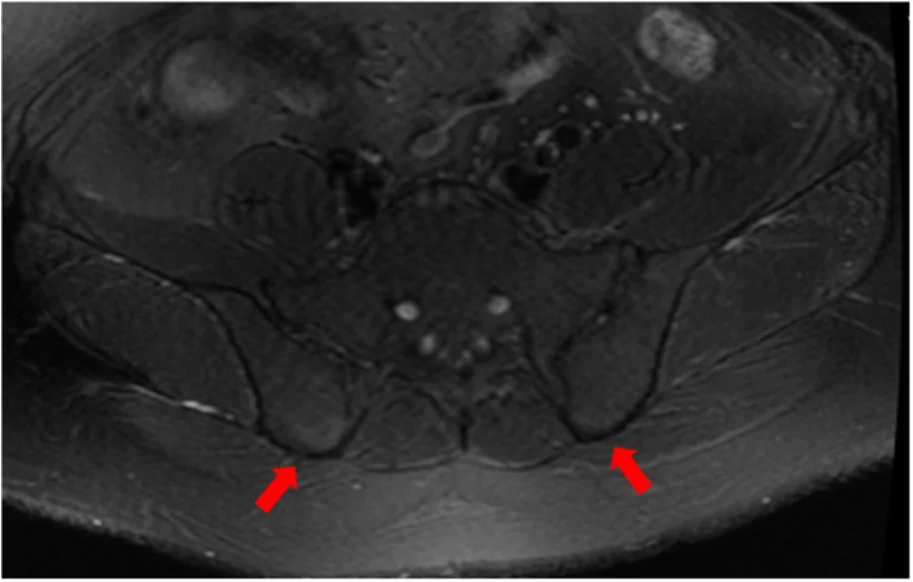
MRI imaging demonstrated bilateral sacroiliitis. (arrows).

In June 2024, laboratory tests revealed persistent inflammation, with an ESR of 45 mm/h (normal < 15 mm/h) and CRP of 10.72 mg/L (normal < 8 mg/L). Computed tomography (CT) (Figure 2) and magnetic resonance imaging (MRI) of the sacroiliac joints (Figure 3) demonstrated bilateral sacroiliitis, prompting a revision of the diagnosis to ankylosing spondylitis (AS). The Ankylosing Spondylitis Disease Activity Score (ASDAS) was calculated as 3.06 (CRP‐based) and 3.25 (ESR‐based), indicating high disease activity. Treatment was switched to ixekizumab (80 mg subcutaneously monthly). Although partial pain relief was achieved, disease control remained suboptimal with persistent inflammatory activity, and analgesic medications could not be discontinued. Given the patient's underlying metabolic syndrome, a JAK inhibitor was not initially considered.

**Figure 4:**
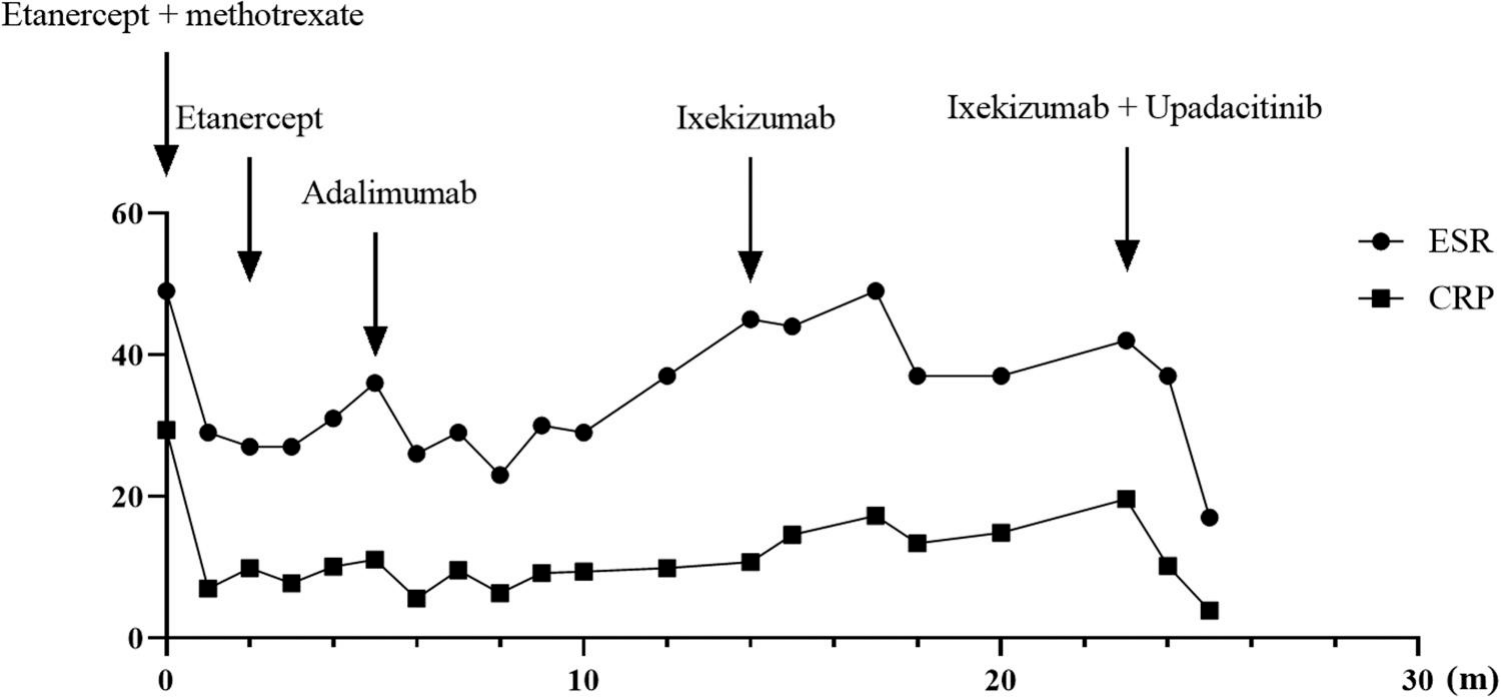
The patient's clinical course, including ESR levels, CRP levels and therapeutic regimens. Month 0 refers to April 2023.

In August 2024, the patient developed pitting nail changes. Upon repeated and detailed questioning prompted by the new nail findings, with family member corroboration, the patient gradually recalled a remote diagnosis of psoriasis during childhood, which had been completely quiescent for over two decades without active cutaneous manifestations. Based on these findings, the diagnosis was revised to PsA according to the 2006 CASPAR classification criteria. Given the suboptimal response to multiple biologic therapies, upadacitinib (15 mg orally once daily) was added to the ongoing ixekizumab regimen. This combination therapy resulted in rapid clinical improvement, with complete resolution of articular symptoms. Follow‐up laboratory tests showed normalization of CRP and ESR levels, while disease activity scores improved significantly (ASDAS‐CRP 0.94; ASDAS‐ESR 1.2), indicating low disease activity. The patient's clinical course, including ESR levels, CRP levels and therapeutic regimens, is shown in Figure 4. To date, the patient has exhibited no adverse reactions. Furthermore, post‐treatment metabolic parameters, including blood pressure, blood glucose, lipid profile, and serum uric acid levels, demonstrated significant improvement (Supporting Information Table S1).

## 4 Discussion

In this study, we present a compelling case of PsA successfully managed through a therapeutic combination of ixekizumab, an IL‐17 inhibitor and upadacitinib, a selective Janus kinase (JAK) inhibitor. The patient, who had previously demonstrated inadequate responses to both tumor necrosis factor (TNF) inhibitors and IL‐17 inhibitor monotherapy, achieved significant disease control following the administration of this dual‐targeted approach.

The patient's diagnostic trajectory—from seronegative rheumatoid arthritis (RA) to ankylosing spondylitis (AS) and ultimately to psoriatic arthritis (PsA)—warrants careful discussion, as this sequence reflects both the inherent heterogeneity of PsA and the diagnostic challenges posed by its incomplete phenotypic expression at presentation. At the initial evaluation in April 2023, the patient presented with progressive polyarthralgia involving the small joints of the hands, wrists, and bilateral hips, accompanied by elevated acute‐phase reactants (ESR 49 mm/h, CRP 29.4 mg/L) and negative serology for rheumatoid factor, anti‐CCP antibodies, and HLA‐B27. Joint ultrasonography confirmed effusion and synovial hyperplasia in the hand and wrist joints. At that time, no active psoriatic skin lesions or nail changes were identified. Crucially, when specifically questioned about any history of skin disease at baseline, the patient did not recall or report a prior diagnosis of psoriasis, as his cutaneous manifestations had resolved completely over 20 years ago without recurrence or residual scarring. Consequently, the presentation fulfilled the 2010 ACR/EULAR classification criteria for seronegative RA [10], and this initial diagnosis was clinically reasonable.

The revision to AS in June 2024 was prompted by CT and MRI evidence of bilateral sacroiliitis, supported by high disease activity scores (ASDAS‐CRP 3.06, ASDAS‐ESR 3.25). Chronic inflammatory back pain and morning stiffness had been present since the initial presentation but were initially attributed to RA‐related cervical or shoulder girdle involvement, or to mechanical pain secondary to obesity and metabolic syndrome. Although HLA‐B27 negativity is atypical in AS—approximately 90% of AS patients are HLA‐B27‐positive—its absence does not exclude the diagnosis [11, 12]. However, the HLA‐B27‐negative status should have prompted earlier consideration of alternative diagnoses within the spondyloarthritis spectrum, particularly PsA, given the known lower HLA‐B27 prevalence in PsA (20–30%) compared with AS (80%–90%) [13, 14]. This serological finding, in retrospect, represented a subtle but important clue that the axial involvement might be better classified as psoriatic spondylitis rather than primary AS. For patients with HLA‐B27 negativity and inflammatory back pain, particularly those with concurrent peripheral arthritis or metabolic syndrome, clinicians should maintain heightened vigilance for PsA and pursue exhaustive screening for occult psoriasis, even when skin lesions are not apparent. Therefore, the HLA‐B27‐negative status in this patient was not merely an atypical feature of AS, but an underappreciated diagnostic red flag that was unrecognized until the characteristic PsA features emerged.

The definitive diagnosis of PsA was established in August 2024 following the emergence of pitting nail changes and, crucially, the elicitation of a history of psoriasis diagnosed more than 20 years prior. Upon repeated and detailed questioning prompted by the new nail findings, with family member corroboration, the patient gradually recalled a remote diagnosis of psoriasis during childhood, which had been completely quiescent for over two decades. Retrospective application of the 2006 CASPAR criteria confirmed the diagnosis: inflammatory articular disease (1 point) + history of psoriasis (2 points) + current nail dystrophy (1 point), yielding a total score of 4 points (≥ 3 required) [15]. This case illustrates that patients with long‐dormant psoriasis may not spontaneously report or even recall their skin disease history, particularly when no active lesions or scarring are present. Therefore, in patients with seronegative inflammatory arthritis, clinicians must employ persistent, structured inquiry regarding remote or childhood skin conditions, supplemented by examination of nails and occult skin sites (scalp, umbilicus, intergluteal cleft), to avoid diagnostic delay.

This diagnostic odyssey highlights the overlapping phenotypic spectra of seronegative RA, axial spondyloarthritis, and PsA, and emphasizes that PsA may sequentially mimic these conditions before its characteristic features become apparent. The repeated treatment modifications necessitated by diagnostic uncertainty further illustrate the clinical consequences of delayed PsA recognition. Notably, the 20‐year history of psoriasis was identified only after 16 months of treatment, suggesting a critical demographic omission that substantially contributed to the diagnostic delay.

From a systemic perspective, this case exposes a critical blind spot in current diagnostic paradigms for seronegative inflammatory arthritis. Neither the 2010 ACR/EULAR RA classification criteria nor the ASAS axial spondyloarthritis criteria incorporate structured screening for remote or dormant psoriasis, creating a diagnostic blind spot for PsA presenting without concurrent skin disease. Our experience suggests that rheumatologists should adopt a “PsA‐first” screening mindset in all patients with seronegative inflammatory arthritis, particularly those with additional features suggestive of the spondyloarthritis spectrum (inflammatory back pain, enthesitis, dactylitis, nail changes, or metabolic syndrome). We propose a standardized screening protocol comprising: directed history‐taking regarding childhood or remote skin conditions (with family member verification when patient recall is unreliable), systematic examination of nails and occult skin sites, and low‐threshold imaging of sacroiliac joints. Furthermore, this case underscores the necessity of enhanced interdisciplinary communication between dermatology and rheumatology; electronic health record systems should ideally flag historical psoriasis diagnoses to alert rheumatologists evaluating inflammatory arthritis, and patients with long‐standing psoriasis should receive periodic rheumatologic screening even after cutaneous remission. The 16‐month diagnostic delay in this case may have allowed irreversible structural damage to accrue, highlighting the urgency of accurate early diagnosis and the potential value of combination therapy as a rescue strategy for refractory disease following diagnostic delay.

The concept of multi‐target therapy for inflammatory arthritis is not new—clinicians began exploring such strategies over 20 years ago. Early attempts in RA, such as combining etanercept with anakinra or etanercept with abatacept, yielded limited success. These regimens were eventually discontinued due to insufficient efficacy and an elevated risk of severe infections [16, 17]. Unlike RA, PsA involves distinct pathogenic mechanisms and presents greater clinical management challenges. Notably, PsA leads to poorer functional outcomes earlier in the disease course, underscoring the need for timely and precisely targeted interventions [5].

Multi‐target therapy in PsA has emerged as a promising strategy to address the complex and heterogeneous nature of the disease, which involves multiple inflammatory pathways. ABT‐122, a dual variable domain immunoglobulin targeting both TNF and IL‐17, was developed to simultaneously inhibit two key cytokines implicated in PsA pathogenesis. However, its efficacy was not significantly differentiated from that of adalimumab, leading to the discontinuation of its development for PsA [18, 19, 20]. Despite this setback, the concept of multi‐target therapy remains relevant. Recent advancements have explored combining b/tsDMARDs with different mechanisms of action to enhance therapeutic outcomes (Table 1). The rationale for such combination therapies lies in the multifaceted immunopathogenesis of PsA. By targeting multiple pathways simultaneously, this approach may allow for reduced drug dosages, mitigate the risk of “escape mechanisms,” and advance personalized treatment strategies [7].

**Table 1:** Combination therapy of biologics or targeted synthetic disease‐modifying anti‐rheumatic drugs in PsA patients.

The first author and year of publication	Age(y.o)/sex	Treatment duration	Joint involvement	Combination regimen	Clinical response	Adverse events
Kitamura 2009 [21]	47/Female	18 months	Peripheral	Efalizumab + Etanercept	Improvement	Tuberculosis
Cuchacovich 2012 [22]	38/Male	11 months	Axial + Peripheral	Ustekinumab + Etanercept	CPDAI improved (15–4)	Increased lipid profile
Heinecke 2013 [23]	23/Female	Not reported	Not reported	Adalimumab + Ustekinumab	BSA improved 80%	Furuncles and autoimmune hemolytic anemia
Babalola 2015 [24]	62/Male	5 months	Axial + Peripheral	Etanercept + Ustekinumab	Improvement	Unstable angina
Gniadecki 2016 [25]	67/Male	62 months	Axial + Peripheral	Ustekinumab + Etanercept	PASI 18.2 → < 1.8, VAS 7.2 → < 5, no swollen joints	Flares of herpes zoster
Gniadecki 2016 [25]	39/Male	50 months	Peripheral	Ustekinumab + Etanercept	PASI 11.2 → 1.6, VAS < 1	Retro tonsillar abscess
Gniadecki 2016 [25]	49/Female	7 months	Peripheral	Ustekinumab + Golimumab	PASI 30 → 1.2, VAS 5.4 → ≤ 2	Skin infection on the lower leg
Gniadecki 2016 [25]	44/Female	18 months	Peripheral	Ustekinumab + Adalimumab	PASI 17.1 → 0‐3, VAS 8 → < 3	No
Gabriele De Marco 2018 [26]	41/Female	15 months	Peripheral	Etanercept + Ustekinumab	Improvement	Developed severe skin infection
Gabriele De Marco 2018 [26]	50/Female	6 months	Peripheral	Ustekinumab + Certolizumab pegol	Improvement	Two chest infections
Gabriele De Marco 2018 [26]	63/Female	3 months	Axial + peripheral	Ustekinumab + Adalimumab	Not reported	Respiratory failure with recurrent chest infections
Gabriele De Marco 2018 [26]	32/Male	24 months	Peripheral	Etanercept + Ustekinumab	Improvement	Developed chest sarcoidosis mediastinal and hilar lymphadenopathy
Gabriele De Marco 2018 [26]	49/Female	15 months	Peripheral	Ustekinumab + Certolizumab Pegol	secondary non‐response joints followed by bout of uveitis	No
Gabriele De Marco 2018 [26]	45/Male	12 months	Axial + peripheral	Ustekinumab + Certolizumab pegol	Lack of significant response from skin and joints	No
Rathod 2019 [27]	46/Female	6 months	Not reported	Adalimumab + Guselkumab	PASI improved (42–2)	No
Thibodeaux 2019 [28]	38/Female	15 months	Peripheral	Etanercept + Guselkumab	Improvement	No
Takamura 2020 [29]	56/Male	6 months	Not reported	Apremilast + Adalimumab	PASI improved (6.4–4.4)	Diarrhea
Takamura 2020 [29]	54/Male	6 months	Not reported	Apremilast + Adalimumab	PASI remained unchanged (3.8–3.8)	Weight loss (> 5% bodyweight
Takamura 2020 [29]	39/Male	6 months	Not reported	Apremilast + Ustekinumab	PASI improved (1.0–0.1)	Diarrhea
Haberman 2021 [30]	27/Female	14 months	Axial + Peripheral	Ustekinumab + Adalimumab	BSA improved (20% to almost full clearance)	One episode of COVID‐19 infection which resolved without treatment
Kashlan 2021 [31]	47/Male	3 months	Axial + Peripheral	Brodalumab + Guselkumab	Improvement	Paradoxical psoriatic arthritis flare
Megan Shurey 2022 [32]	56/Male	17 months	Peripheral	Tofacitinib + Risankizumab	BSA improved (2%–1%)	No
Megan Shurey 2022 [32]	36/Female	17 months	Peripheral	Tofacitinib + Guselkumab	PASI improved (9.6 to BSA < 1%)	No
Megan Shurey 2022 [32]	54/Female	11 months	Peripheral	Tofacitinib + Ustekinumab	No improvement in swollen joint count Decreased joint pain; BSA improved (> 3%–< 1%)	No
Megan Shurey 2022 [32]	70/Female	11 months	Peripheral	Tofacitinib + Ixekizumab	No improvement in swollen joint count Decreased joint pain; BSA remained cleared (0%–< 1%)	No
Megan Shurey 2022 [32]	45/Female	11 months	Axial + peripheral	Tofacitinib + Ixekizumab	BSA improved (small patch scalp and ear to < 1%)	One episode of uveitis
Megan Shurey 2022 [32]	57/Male	6 months	Peripheral	Tofacitinib + Secukinumab	BSA improved (90%–0%)	C. difficile recurrence
Cristina Valero 2022 [33]	60/Female	8 months	Axial+ Peripheral	Secukinumab + Etanercept	ASDAS‐CRP improved (4.6–2.1)	No
Cristina Valero 2022 [33]	65/Female	6 months	Peripheral	Ixekizumab + Adalimumab	DAS‐28‐CRP improved (3.9–1.4)	No
Cristina Valero 2022 [33]	62/Male	3 months	Peripheral	Secukinumab + Adalimumab	DAS‐28‐CRP worsened (4.4–4.51)	No
Cristina Valero 2022 [33]	60/Female	14 months	Axial + Peripheral	Golimumab + Secukinumab	DAS‐28‐CRP improved (5.0–2.8)	No
Hanna 2022 [34]	49/Male	12 months	Peripheral	Golimumab + Risankizumab	PASI improved (24.6–0)	No
Zimmer 2022 [35]	62/Male	28 months	Peripheral	Apremilast + Etanercept	PASI improved (10–5)	No
Grace Hren 2024 [36]	50 s/Female	930 days	Peripheral	Upadacitinib + Ixekizumab	MDA achieved at day 275	No
Grace Hren 2024 [36]	40 s/Female	56 days	Peripheral	Upadacitinib + Guselkumab	MDA achieved at day 56	No
Grace Hren 2024 [36]	40 s/Male	301 days	Peripheral	Upadacitinib + Brodalumab	MDA achieved at day 169	No
Grace Hren 2024 [36]	70 s/Female	338 days	Axial + peripheral	Deucravacitinib + Ixekizumab	MDA achieved at day 89, sustained for 249 days, then PSO flared	No
Grace Hren 2024 [36]	50 s/Female	260 days	Axial + peripheral	Deucravacitinib + Ixekizumab	MDA achieved at day 42, sustained for 218 days then PSO flared	Bronchitis
Grace Hren 2024 [36]	50 s/Male	44 days	Peripheral	Upadacitinib + Guselkumab	MDA not yet achieved at day 44	No
Current study	35/Male	10 months	Axial + peripheral	Ixekizumab + Upadacitinib	ASDAS‐CRP improved (3.06–0.94), ASDAS‐ESR improved (3.25–1.2)	No

Abbreviations: ASDAS‐CRP, Ankylosing Spondylitis Disease Activity Score‐C‐reactive protein; ASDAS‐ESR, Ankylosing Spondylitis Disease Activity Score‐erythrocyte sedimentation rate; BSA, body surface area; CPDAI, Composite Psoriatic Disease Activity Index; DAS‐28‐CRP, Disease Activity Score‐28‐C‐reactive protein; MDA, minimal disease activity; PASI, Psoriasis Area and Severity Index; PsA, psoriatic arthritis; PSO, psoriasis; VAS, visual analog scale.

The use of combination b/tsDMARD therapy in psoriatic arthritis necessitates heightened clinical vigilance for infectious complications, given the potential for synergistic immunosuppression. Although our case revealed no significant infectious adverse events, published case reports have identified bacterial and viral infections associated with such therapeutic combinations [32]. These clinical observations warrant systematic surveillance in patients receiving concurrent biologic therapies. Nevertheless, the precise quantification of infection risk attributable to combination therapy in PsA patients requires validation through large‐scale, controlled clinical trials with extended follow‐up periods.

Moreover, our patient presented with comorbid metabolic syndrome (MetS). Importantly, a bidirectional relationship exists between PsA and MetS, mediated by shared pathophysiological mechanisms including chronic inflammation, insulin resistance, and dysregulated adipokine secretion [37, 38]. Clinical evidence demonstrates that PsA patients with MetS exhibit higher disease activity, greater functional impairment, and reduced responsiveness to conventional therapies compared to those without metabolic comorbidities [39, 40]. These observations suggest that patients with PsA and concomitant MetS may particularly benefit from multi‐target combination therapy approaches. The metabolic improvements observed in our patient following combination therapy with ixekizumab and upadacitinib warrant specific consideration of the JAK inhibitor component. Upadacitinib, a selective JAK1 inhibitor, has been associated with dose‐dependent increases in lipid levels in clinical trials [41]; however, our patient demonstrated paradoxical lipid improvement. This favorable metabolic profile likely reflects the dominant anti‐inflammatory effect of combination therapy overriding the drug's intrinsic lipid‐raising potential, as suppression of systemic inflammation improved insulin sensitivity and lipid clearance. Notably, our patient achieved substantial weight reduction during the treatment course, and this weight loss likely contributed independently to the metabolic improvements, particularly the reductions in blood pressure, fasting glucose, and uric acid levels, unrelated to drug‐specific effects. The interplay between effective disease control, restored physical activity, and potential JAK inhibitor–associated effects on appetite or metabolism may have collectively facilitated this favorable outcome.

### 4.1 Patient Perspective

It took over a year and several diagnoses before I learned I had psoriatic arthritis. Each new treatment helped only a little, and I could not stop my painkillers, which was frustrating. After the second medicine was added, my joint pain and morning stiffness disappeared within weeks. I feel healthier than I have in years.

### 4.2 Study Limitations

Several limitations of this study should be acknowledged. First, as a single‐case report, our findings are inherently limited in generalizability and cannot establish causality or predict response in the broader PsA population. Second, although the follow‐up duration was sufficient to demonstrate initial clinical efficacy, it remains relatively short for fully assessing long‐term safety outcomes, particularly regarding infection risk, cardiovascular events, and malignancy associated with dual immunosuppression. Third, while our scoping review was comprehensive in mapping existing evidence, it was limited to PubMed and did not include other databases. Fourth, publication bias may favor positive outcomes in case reports, potentially overestimating the efficacy and underestimating the adverse events of combination therapy.

### 4.3 Future Directions

Our findings highlight several avenues for future investigation. First, prospective randomized controlled trials are urgently needed to formally evaluate the efficacy and safety of IL‐17/JAK inhibitor combination therapy in PsA, with predefined endpoints encompassing joint, skin, axial, and entheseal domains, as well as patient‐reported outcomes. Second, biomarker‐driven studies should seek to identify immunological or genetic signatures that predict response to dual‐target therapy, thereby enabling precision selection of candidates for combination treatment rather than empirical use. Third, long‐term observational studies and registry data are essential to quantify the incidence of serious infections, cardiovascular events, venous thromboembolism, and malignancy in patients receiving concurrent b/tsDMARDs, as the current evidence base remains confined to short‐term case reports. Fourth, treatment de‐escalation trials are warranted to determine whether durable remission can be maintained with reduced dosing or sequential monotherapy after initial combination‐induced disease control.

## 5 Conclusion

This case report highlights the potential of IL‐17/JAK inhibitor combination therapy for refractory PsA, demonstrating rapid clinical improvement in a treatment‐resistant patient. While promising for addressing PsA's complex pathogenesis, combination b/tsDMARDs require rigorous safety monitoring due to infection risks. Current evidence remains limited to case reports, underscoring the need for controlled trials to establish efficacy, safety, and optimal patient selection criteria before clinical adoption.

## Author Contributions

The case was diagnosed and followed up by Qi Zhang and Min Zhao. Literature search and summarization were performed by Mengduan Pang, Ling Li and Min Zhao. Mengduan Pang wrote the article.

## Funding

The authors have nothing to report.

## Consent

Written informed consent has been obtained from the patient to publish this article.

## Conflicts of Interest

The authors declare no conflict of interest.

## Supporting information


Supporting File

## Data Availability

The data that support the findings of this study are available from the corresponding author upon reasonable request. The analyzed data sets generated during the study are available from the corresponding author upon reasonable request.
