# Supplementary material for: Combination Treatment of IL‐17 Inhibitor and JAK Inhibitor in Psoriatic Arthritis: A Case Report and Scoping Review
Source: Immun Inflamm Dis. 2026 Sep 11;14(9):e70515. doi: 10.1002/iid3.70515 (PMC13564248; doi:10.1002/iid3.70515)
Supplement: Supplementary file 1 [file IID3-14-e70515-s001.docx]

| Parameter | Baseline (April 2023) | Pre-combination (August 2024) | Post-combination (June 2025) | Normal range |
| --- | --- | --- | --- | --- |
| Body weight (kg) | 105 | 103 | 95 | - |
| BMI (kg/m²) | 31.7 | 31.1 | 28.7 | 18.5 - 23.9 |
| Systolic BP (mmHg) | 143 | 142 | 137 | <140 |
| Diastolic BP (mmHg) | 97 | 98 | 82 | <90 |
| Fasting glucose (mmol/L) | 8.88 | 8.32 | 6.89 | 3.9 - 6.1 |
| HbA1c (%) | 6.8 | 6.9 | 6.5 | <6.5 |
| Total cholesterol (mmol/L) | 6.31 | 6.11 | 5.00 | <5.2 |
| Triglycerides (mmol/L) | 5.03 | 6.72 | 4.9 | <1.7 |
| LDL-C (mmol/L) | 3.40 | 3.24 | 2.9 | <3.4 |
| HDL-C (mmol/L) | 0.92 | 0.86 | 0.86 | >1.0 |
| Serum uric acid (μmol/L) | 689 | 456 | 396 | 208 - 428 |
| ALT (U/L) | 55 | 39 | 41 | <50 |

SUPPLEMENTARY TABLE 1 ǀ Metabolic parameters before and after combination therapy
